# Efficacy of problem-based learning approach for teaching evidence-based practice to midwives and nurses: A systematic review protocol

**DOI:** 10.18332/ejm/215324

**Published:** 2025-12-31

**Authors:** Grace Komuhangi, Florian Neuhann, Valerie R. Louis, Moses Ocan, Alison Annet Kinengyere, Jürgen Wacker

**Affiliations:** 1 Heidelberg Institute of Global Health, University Hospital and Medical Faculty, Heidelberg University, Heidelberg, Germany; 2 Department of Pharmacology and Therapeutics, College of Health Sciences, Makerere University, Kampala, Uganda; 3 Albert Cook Library, College of Health Sciences, Makerere University, Kampala, Uganda; 4 Department of Obstetrics and Gynecology, Faculty of Medicine, Heidelberg University, Heidelberg, Germany

**Keywords:** problem-based learning, evidence-based practice, nursing education, midwifery education, systematic review

## Abstract

Problem-Based Learning (PBL) has emerged as a promising educational approach for developing Evidence-Based Practice (EBP) competencies in nursing and midwifery education. PBL is a student-centered educational approach that uses authentic, ill-structured clinical problems as the starting point for learning, where small groups of students work collaboratively under facilitator guidance to identify learning objectives and apply knowledge to solve real-world problems. However, there is limited synthesized evidence on PBL's effectiveness specifically for teaching EBP to nursing and midwifery professionals globally. This systematic review aims to evaluate the efficacy of PBL approaches in teaching EBP to nursing and midwifery students and professionals. A comprehensive search will be conducted in MEDLINE, CINAHL, PubMed, EMBASE, Web of Science, ERIC, PsycINFO, and Cochrane CENTRAL, covering studies from 2001 to October 2024. Studies will be included if they evaluate PBL interventions for teaching EBP to nursing or midwifery students or professionals. Two independent reviewers will screen studies, extract data, and assess methodological quality using JBI-SUMARI tools. Due to anticipated heterogeneity, narrative synthesis will be the primary approach, with meta-analysis conducted if sufficient homogeneity exists. This review will provide evidence on PBL's effectiveness for EBP education and inform curriculum development and educational policy in nursing and midwifery programs globally.

## INTRODUCTION

### Rationale and context

For many years, midwifery education has transitioned to higher education, where principles of evidence-based practice should be integrated into the curriculum^1^. The shift from apprenticeship-based to academic education models in nursing and midwifery has fundamentally shaped how Evidence-Based Practice (EBP) is integrated into professional curricula^2^. EBP represents a systematic approach to clinical decision-making that integrates the best available research evidence with clinical expertise and patient values to optimize healthcare outcomes^3,4^.

Midwives must have EBP knowledge and skills in order to use a high level of professional judgment, clinical reasoning, and decision making^5,6^. As a result, in order to ensure the quality of midwifery practice, EBP concepts must be implemented through effective pedagogical approaches, so that future midwives can learn to conduct research and apply the best available evidence in practice^7,8^.

With the dynamic and complex nature of today’s healthcare work environment, midwifery educators face an increasing number of challenges in ensuring that undergraduate midwifery students possess the necessary knowledge, skills, and attitudes for competent patient care, as well as the capacity to adapt to change^9^. Additionally, the process of learning and teaching evidence-based practice (EBP) provides significant hurdles for undergraduate students and educators alike, as just supplying students with knowledge does not guarantee that students would feel capable of practicing EBP in their final clinical settings^10,11^.

Accreditation guidelines for entry-level midwifery and nursing programs all anticipate that graduates will possess the necessary abilities and competences to solve clinical problems autonomously^1^. Fraser and Greenhalgh^12^ defined competence as what individuals know or are able to do in terms of knowledge, skills and attitudes.

Despite the growing interest in PBL for EBP education, there remains limited synthesis of the global evidence on its effectiveness, particularly in the context of midwifery and nursing education^11,13^. While several individual studies and localized evaluations have been conducted, a comprehensive and systematic review is needed to assess the efficacy of the PBL approach in enhancing EBP competencies among midwifery and nursing students and professionals^10,11^. Moreover, although both nursing and midwifery education face challenges in integrating EBP, midwifery programs are uniquely influenced by their emphasis on physiological birth, continuity-of-care models, and relational, community-embedded practice, which may require pedagogical approaches distinct from those traditionally applied in nursing^13^.

### Existing evidence on PBL in healthcare education

While systematic reviews in related healthcare fields have shown promising results for PBL effectiveness in medical education, the translation of these findings to nursing and midwifery contexts remains unclear^11,13^. Preliminary evidence suggests that PBL may be more effective than traditional lecture-based methods for developing critical thinking and clinical reasoning skills, but comprehensive synthesis specific to nursing and midwifery EBP education is lacking^11,13^.

### Gap in the literature

Existing reviews on EBP education have largely focused on general teaching strategies or have combined data across multiple health professions, often without differentiating between pedagogical approaches or disciplinary contexts^11,13^. Moreover, few reviews have examined the impact of PBL on specific EBP outcomes, such as knowledge acquisition, attitude change, skill development, and self-efficacy, within midwifery and nursing education^10,11^. Given the unique educational needs, clinical environments, and scopes of practice of these professions, tailored evidence is necessary to inform curriculum design, teaching methods, and faculty development^1,8^.

### Evidence gap

Modern teaching strategies such as Problem Based Learning (PBL) aim to promote cognitive skills among undergraduate healthcare professionals^9,14^. While previous systematic reviews have evaluated PBL’s effectiveness in general healthcare education and postgraduate medical training, none has specifically examined PBL’s efficacy for teaching EBP to nursing and midwifery professionals^11,13^. This represents a significant knowledge gap given that nursing and midwifery educational contexts differ substantially from medical education in curriculum structure, learning objectives, and professional scope^2,9^.

### Definition of problem-based learning

Problem-Based Learning (PBL) is defined as a student-centered educational approach that uses authentic, ill-structured problems (complex and ambiguous) as the starting point for learning, where small groups of students work collaboratively under facilitator guidance to identify learning objectives, engage in self-directed study, and apply knowledge to solve real-world problems^3,14^.

*Core characteristics of PBL for the review*

For the purposes of the systematic review, PBL interventions must demonstrate the following essential characteristics. Problem-Based Learning (PBL) progresses through seven systematic steps that guide learners from initial problem exploration to application of new knowledge. First, students clarify unfamiliar terms within the scenario to build a shared understanding. Second, they identify and define the core problem that needs to be addressed. Third, learners brainstorm possible explanations or hypotheses, activating prior knowledge without evaluating accuracy. In the fourth step, the group organizes and structures these ideas, clustering them to expose knowledge gaps. This leads to step five, where they formulate learning objectives that direct their inquiry. Step six involves self-directed learning, where students individually or collaboratively seek evidence from literature and other resources, an approach foundational to the original PBL model in medical education^1^. Finally, in step seven, learners synthesize, discuss, and apply their new knowledge, revisiting their hypotheses and reflecting on how the insights translate into practice, a key element in preparing professionals to navigate complex real-world contexts^2^.

*PBL process for EBP education*

In the context of Evidence-Based Practice education, PBL typically follows this process:

- Step 1. Presentation and clarification of the clinical scenario. Students are presented with a clinical scenario that requires evidence-based decision-making, and they clarify unfamiliar terms or concepts embedded in the case.
- Step 2. Problem identification and definition. The group analyses the scenario to identify the core EBP-related problems, specifying learning issues connected to EBP competencies such as evidence retrieval, appraisal, and application.
- Step 3. Brainstorming of prior knowledge and possible explanations. Students activate prior knowledge by discussing what they already understand about EBP principles, clinical uncertainty, or possible decision pathways.
- Step 4. Structuring and organizing ideas. The group categorizes their ideas and identifies gaps in their EBP knowledge, for example, gaps in search skills, appraisal criteria, or guideline use.
- Step 5. Formulation of learning objectives. Learners transform the identified gaps into specific learning objectives (e.g. identify appropriate databases, apply critical appraisal tools, interpret evidence levels).
- Step 6. Self-directed learning. Students independently study EBP principles, search strategies, clinical guidelines, methodological quality tools, and other relevant resources to meet their learning objectives.
- Step 7. Synthesis, application, and reflection. The group reconvenes to share and discuss what they have learned, apply EBP knowledge to resolve the clinical problem, and reflect on both the learning process and learning outcomes.

### Distinction from other teaching methods

PBL differs from traditional lecture-based learning by placing problems before theory, from case-based learning by emphasizing self-directed inquiry over instructor-led discussion, and from simulation-based learning by focusing on cognitive rather than psychomotor skill development^9,10^.

*Relevance of PBL for EBP education*

PBL is particularly well-suited for EBP education because it mirrors the EBP process itself: clinical problems trigger questions, questions drive evidence searching, evidence requires critical appraisal, and findings must be integrated into clinical decision-making. This alignment makes PBL a theoretically coherent approach for developing EBP competencies^9,10^.

*Challenges in PBL implementation*

Despite its potential benefits, PBL implementation in nursing and midwifery education faces several challenges:

- Faculty readiness and training: PBL requires skilled facilitators who can guide discussions without providing direct instruction, necessitating comprehensive faculty development programs.
- Student adaptation: Some students may struggle with the self-directed nature of PBL, especially those accustomed to traditional learning methods.
- Resource intensity: PBL may demand more time, infrastructure, and academic resources compared to conventional lecture-based approaches.

### Objectives

*Primary objective*

The primary objective is to systematically evaluate the efficacy of Problem-Based Learning approaches for teaching Evidence-Based Practice to nursing and midwifery students and professionals globally, compared to other educational methods or no intervention.

*Secondary objectives*

The secondary objectives include:

- Effectiveness assessment: To determine the comprehensive impact of on specific Evidence-Based Practice competencies, including knowledge of EBP principles and processes, attitudes toward evidence-based care, and development of critical skills in clinical question formulation, literature searching, and critical appraisal.
- Intervention characterization: To identify and describe the key characteristics of PBL interventions specifically designed for EBP education, examining duration, intensity, format, facilitator training requirements, curriculum integration, and assessment methods.
- Comparative analysis: To establish the relative effectiveness of PBL against other established teaching approaches commonly used in healthcare education, including traditional lecture-based instruction, case-based learning methodologies, simulation-based education programs, and online or blended learning methods.
- Implementation factors: To investigate contextual elements influencing PBL effectiveness for EBP education, considering student characteristics, institutional factors, cultural and geographical contexts, and barriers and facilitators that affect successful PBL implementation.
- Evidence synthesis: To translate research findings into practical, evidence-based recommendations for educational practice and policy, identifying optimal PBL implementation strategies for EBP education and establishing future research priorities.

## METHODS

### Protocol registration and reporting guidelines

This systematic review protocol follows the PRISMA-P (Preferred Reporting Items for Systematic Reviews and Meta-Analyses Protocols)^15^ reporting guidelines as the guiding framework and has been registered with the International Prospective Register of Systematic Reviews (PROSPERO: CRD42023390989). The completed systematic review will be reported according to PRISMA 2020 guidelines^16^.

### Research questions

The primary research question of our review is: ‘What is the efficacy of Problem-Based Learning approaches for teaching Evidence-Based Practice to nursing and midwifery students and professionals compared to other educational methods?’. Specific research questions include: 1) ‘How effective is PBL compared to other teaching methods in improving EBP knowledge, attitudes, skills, and behaviors among nursing and midwifery learners?’; 2) ‘Which specific EBP competencies are most effectively developed through PBL interventions?’; 3) ‘What are the key characteristics of successful PBL interventions for EBP education?’; 4) ‘What factors influence the effectiveness of PBL for EBP education in nursing and midwifery programs?’; and ‘What is the long-term impact of PBL-based EBP education on clinical practice behaviors?’.

### Eligibility criteria

Eligibility criteria include:

- Population: Nursing students (undergraduate, diploma, associate degree, baccalaureate) and midwifery students (pre-registration, undergraduate, graduate) enrolled in formal educational programs, and practicing nurses and midwives participating in continuing professional development or post-qualification EBP education programs.
- Intervention: Problem-Based Learning interventions specifically designed to teach or include Evidence-Based Practice competencies that meet all essential PBL characteristics and explicitly address one or more EBP competencies.
- Comparison: Other educational approaches including traditional lecture-based instruction, case-based learning, simulation-based education, online learning modules, seminar-based discussions, experiential learning, or control groups (wait-list controls, no-intervention controls, or standard curriculum groups).
- Outcomes: Primary outcomes focus on the efficacy and implications of PBL for EBP among nursing and midwifery professionals, including EBP knowledge acquisition, attitudes toward evidence-based care, critical appraisal skills, clinical decision-making abilities, research literacy, and long-term application of EBP competencies in clinical practice. Secondary outcomes include cognitive skills development, self-efficacy in EBP application, and educational satisfaction.
- EBP assessment tools: To ensure consistency and comparability, we will prioritize studies using validated, standardized EBP assessment tools including Evidence-Based Practice Questionnaire (EBPQ)^17^, Evidence-Based Practice Beliefs Scale (EBPB)^18^, Student Evidence-Based Practice Questionnaire (S-EBPQ)^19^, Fresno Test of Competence in Evidence-Based Medicine^20^, and the ACE Tool (Assessing Competency in Evidence-Based Practice)^21^. Studies using other valid assessment tools will be included but analyzed separately in sensitivity analyses to assess the impact of measurement variability on outcomes.
- Study design: Randomized controlled trials (RCTs), quasi-experimental studies, controlled before-after studies, cohort studies, and mixed-methods studies will be included. Qualitative studies exploring student and faculty experiences with PBL for EBP learning will be included for thematic synthesis.

### Inclusion criteria

Our inclusion criteria include peer-reviewed studies published from 2001 to August 2025 that evaluate PBL interventions that include EBP education components. Participants must be nursing or midwifery students or professionals. Studies must report quantitative outcomes related to EBP competencies and may include comparative studies with control or comparison groups. Overall, studies must demonstrate all essential PBL characteristics as defined in this manuscript.

Although English-language publications form the primary focus of this review, non-English studies will be included when they meet all other inclusion criteria and when translation is feasible. This approach is adopted to minimize language bias, ensure comprehensive coverage of global PBL-EBP research, and align with international methodological standards for systematic reviews.

Although the primary objective of this review is to assess the efficacy of PBL for teaching EBP, we will also include qualitative studies because they offer essential contextual and explanatory insights into how and why PBL produces its effects. Such evidence complements quantitative outcomes by elucidating learning processes, implementation dynamics, and participant experiences, thereby contributing meaningfully to our overall understanding of efficacy.

### Exclusion criteria

Studies focusing exclusively on other healthcare professionals without nursing/midwifery participants will be excluded, as also studies evaluating PBL for general clinical skills without EBP components, non-peer-reviewed publications, conference abstracts, editorials, or opinion pieces, studies without original data and single-group pre-post studies without controls.

### Information sources

Electronic databases will be searched from January 2001 to August 2025, including MEDLINE (via PubMed), CINAHL, EMBASE, Web of Science, ERIC, PsycINFO, Cochrane CENTRAL, and Google Scholar.

To capture non-English language studies with English abstracts and reduce language bias, we will additionally search: SciELO (Scientific Electronic Library Online) – for Spanish and Portuguese language studies from Latin America and Spain; LILACS (Latin American and Caribbean Health Sciences Literature) – for studies from Latin American countries; CAIRN – for French language studies; BASE (Bielefeld Academic Search Engine) – for German language studies; and CEEOL (Central and Eastern European Online Library) – for studies from Central and Eastern European countries.

Additional sources will include the reference lists of included studies and relevant systematic reviews, hand-searching specific key journals (such as the Journal of Evidence-Based Medicine, Worldviews on Evidence-Based Nursing, Nurse Education Today, Midwifery). Additional sources may include grey literature: ProQuest dissertations and theses, conference proceedings; contacting experts in the field for unpublished or in-press studies; World Health Organization (WHO) databases; International Council of Nurses (ICN) publications; and the International Confederation of Midwives (ICM) resources.

### Study selection process

Study selection will follow a two-stage process:

- Stage 1. Title and Abstract screening: Two independent reviewers will screen all titles and abstracts against the eligibility criteria using Covidence systematic review software. Studies clearly not meeting inclusion criteria will be excluded.
- Stage 2. Full-text review: Full texts of potentially eligible studies will be retrieved and independently assessed by two reviewers. Reasons for exclusion at this stage will be documented.

Disagreements at both stages will be resolved through discussion, and if necessary, consultation with a third reviewer. Inter-rater reliability will be assessed using Cohen’s kappa coefficient. The study selection process will be documented in a PRISMA flow diagram.

### Quality assessment

Methodological quality and risk of bias will be assessed independently by two reviewers using the Joanna Briggs Institute (JBI) Critical Appraisal Tools²². Different JBI tools will be used based on study design. Each study will be rated as low, moderate, or high risk of bias. Studies will not be excluded based on quality assessment, but quality ratings will inform sensitivity analyses and interpretation of findings. Disagreements will be resolved through discussion with a third reviewer if necessary.

### Data extraction

A standardized data extraction form will be developed and piloted on a sample of five studies prior to full implementation. Following this piloting phase, two independent reviewers will extract all relevant information from each included study. For each study, details on the study characteristics will be collected, including the authors, year of publication, and country in which the study was conducted. Information on the study design, setting, funding sources, and any reported conflicts of interest will also be recorded. Reviewers will document the sample size and participant characteristics, such as age, gender, education level, and any prior knowledge of evidence-based practice.

Comprehensive information on the PBL intervention will likewise be extracted. This will include the duration of the intervention in terms of hours, weeks, or months, as well as its intensity, reflected by the number of contact hours per week. The composition and size of student groups will be described, along with details on the facilitators, including their qualifications and any specific training they received. Additional elements of the intervention, such as the nature of the PBL scenarios or problems used, the assessment methods applied, the degree of fidelity to core PBL principles outlined, and whether the intervention was integrated into the curriculum or delivered as a standalone module, will all be carefully captured.

The measurement of outcomes will be described in detail. Reviewers will identify the name and version of each EBP assessment tool used, noting whether the tool is validated and outlining its psychometric properties where available. The specific EBP domains measured, such as knowledge, attitudes, skills, behaviors, or self-efficacy, will be recorded, together with the timing of outcome assessments, whether at baseline, post-intervention, or during follow-up periods. Reported information on the reliability and validity of the tools, along with quantitative results such as effect sizes, means, standard deviations, confidence intervals, and p-values, will be extracted as well.

For studies that include a comparison or control group, the nature of the comparator intervention will be described, including its duration and intensity. In studies that incorporate qualitative components, reviewers will extract information on student and faculty experiences with PBL, the barriers and facilitators encountered during implementation, and contextual factors that may have influenced the effectiveness of the approach.

Where necessary, authors of the included studies will be contacted to clarify missing or unclear data. Any discrepancies in data extraction between the two reviewers will be discussed and resolved in consultation with a third reviewer.

### Search strategy

The search strategy combines three main concept groups using Boolean operators:

- Concept 1. Problem-based learning terms. ‘Problem-based learning’ OR ‘Problem based learning’ OR ‘PBL’ OR ‘Problem-solving learning’ OR ‘Case-based learning’.
- Concept 2. Evidence-based practice terms: ‘Evidence-based practice’ OR ‘Evidence based practice’ OR ‘EBP’ OR ‘Evidence-based nursing’ OR ‘Evidence-based care’.
- Concept 3. Nursing and Midwifery terms: ‘Nursing’ OR ‘Nurse’ OR ‘Midwifery’ OR ‘Midwife’ OR ‘Healthcare education’.

An example search string for PubMed: ((‘problem-based learning’[Title/Abstract] OR ‘problem-based learning’[Title/ Abstract] OR ‘PBL’[Title/Abstract]) AND (‘evidence-based practice’[Title/Abstract] OR ‘evidence-based practice’[Title/ Abstract] OR ‘EBP’[Title/Abstract])) AND (‘nursing’[Title/Abstract] OR ‘midwifery’[Title/Abstract])

The search strategy will be adapted for each database and peer-reviewed through an iterative process (Supplementary file). Search terms will be adapted for different databases using appropriate subject headings (MeSH terms for PubMed, CINAHL headings for CINAHL). Duplicate removal will be conducted using EndNote reference management software with manual verification of potential duplicates.

### Study selection and data collection process

Two independent reviewers will conduct study selection through duplicate removal, title and abstract screening, full-text assessment, and consensus processes. Disagreements will be resolved through discussion or third-party consultation. The selection process will be documented using a PRISMA flow diagram^16^.

Data will be extracted using standardized JBI-SUMARI tools by two independent reviewers. Extracted information will include study characteristics, participant characteristics, intervention details, and outcome measures. Primary authors will be contacted if additional information is required.

When critical data points are missing or unclear, the following procedures will be implemented: primary study authors will be contacted via email with up to two follow-up attempts, if missing data cannot be obtained, sensitivity analyses will be conducted to assess the impact on overall findings. Studies with substantial missing outcome data (>20%) will be excluded from meta-analysis but included in narrative synthesis.

### Risk of bias assessment

Methodological quality will be assessed using appropriate Joanna Briggs Institute (JBI) critical appraisal tools from JBI-SUMARI, including checklists for randomized controlled trials, quasi-experimental studies, analytical cross-sectional studies, and cohort studies. Two independent reviewers will conduct assessments, with disagreements resolved through discussion.

### Rationale for including non-randomized studies

While RCTs represent the gold standard for evaluating intervention efficacy, non-randomized studies provide valuable insights into real-world implementation of PBL in diverse educational settings. Cross-sectional and quasi-experimental studies will be included to capture the breadth of available evidence, but will be weighted differently in the analysis and clearly distinguished in the results. Sensitivity analyses will compare findings with and without observational studies to assess the robustness of conclusions.

### Data synthesis

Given the anticipated heterogeneity in PBL interventions, study designs, populations, and outcome measures, we will employ a comprehensive multi-faceted synthesis strategy that integrates quantitative and qualitative findings.

*Primary approach: narrative synthesis*

Narrative synthesis will be conducted following the framework proposed by Popay et al.^23^, which consists of four iterative elements.

The preliminary synthesis phase will involve creating structured tables to summarize study characteristics, interventions, outcomes, and results through systematic tabulation. Studies will be organized by outcome domain (knowledge, attitudes, skills, behaviors), PBL characteristics (duration, intensity, fidelity), and population characteristics (undergraduate vs postgraduate; nurses vs midwives). Visual representations using harvest plots and effect direction plots will display patterns of effects across studies.

Exploring relationships within and between studies will involve analyzing patterns by intervention characteristics, such as examining whether longer duration or higher intensity PBL leads to better outcomes. We will examine contextual factors including geographical setting, education level, and resource availability, while investigating temporal trends to determine if PBL effectiveness has changed over time. This exploration will also identify moderators and mediators of PBL effectiveness.

Assessment of robustness of synthesis will be conducted through sensitivity analyses by excluding high risk of bias studies. Subgroup analyses will be performed by study design, assessment tool, and population. The strength and consistency of findings across different study characteristics will be carefully evaluated.

Finally, we will formulate evidence-based conclusions about PBL effectiveness for EBP education, considering the strength and quality of evidence, consistency of findings, and applicability to different contexts.

### Complementary synthesis approaches

*Thematic synthesis for qualitative data*

Following the approach of Thomas and Harden^24^, we will conduct thematic synthesis of qualitative findings to complement quantitative results. This process involves line-by-line coding of qualitative findings from included studies, followed by development of descriptive themes by grouping similar codes. We will then generate analytical themes that go beyond the primary studies to produce new interpretive insights.

Specifically, thematic synthesis will identify barriers and facilitators to PBL implementation for EBP education, as well as student and faculty experiences with PBL for EBP learning. We will examine contextual factors influencing the effectiveness of PBL interventions and explore mechanisms through which PBL may enhance or hinder EBP competency development.

*Framework synthesis*

Using the six core PBL characteristics identified as an a priori framework, we will systematically analyze how adherence to or variation from core PBL principles influences educational outcomes. We will determine which specific PBL components, such as small-group collaboration, self-directed learning, and facilitator role, are most critical for EBP education effectiveness. Additionally, we will examine how different combinations or configurations of PBL elements relate to outcome patterns. This framework synthesis will help identify the ‘active ingredients’ of effective PBL interventions for EBP education.

### Managing heterogeneity

Anticipated heterogeneity in interventions, populations, and outcomes will be managed through multiple complementary strategies.

*Detailed characterization of interventions*

All PBL interventions will be systematically characterized using a standardized extraction form capturing duration (total hours, weeks, or months of intervention), intensity (number of contact hours per week), and group size (number of students per PBL group). We will document facilitator qualifications including educational background and PBL training, as well as assessment methods distinguishing between formative versus summative and individual versus group approaches. The degree of curriculum integration, whether standalone module or integrated throughout curriculum, will be recorded. Finally, fidelity to core PBL principles will be assessed through an adherence rating based on the outlined criteria.

*Subgroup analyses*

Pre-specified subgroup analyses will be conducted to explore sources of heterogeneity. These will compare PBL implementation models, contrasting pure PBL (meeting all six core characteristics) with hybrid PBL approaches (meeting 4–5 characteristics). We will examine differences between educational settings, specifically classroom-based PBL versus clinical practice-based PBL. Target populations will be analyzed separately, including undergraduate students versus postgraduate students, pre-registration nurses versus midwives, and practicing nurses/midwives. Geographical regions will be compared, distinguishing between high-income countries and low- and middle-income countries.

*Meta-regression (if sufficient studies)*

If a sufficient number of studies (≥10) with comparable outcomes are identified, random-effects meta-regression will be conducted to explore how intervention characteristics influence effect sizes. Potential moderator variables include total intervention duration (hours), intensity of facilitator training (hours), group size (number of students), degree of curriculum integration (standalone vs integrated), and baseline EBP knowledge level of participants.

*Sensitivity analyses*

Multiple sensitivity analyses will be conducted to assess the robustness of findings. These will include excluding studies with low adherence to core PBL principles (meeting <4 of 6 characteristics), excluding studies not using validated EBP assessment tools, and excluding studies rated as high risk of bias. We will also compare studies published pre-2010 versus post-2010 to explore the evolution of PBL approaches.

### Meta-analysis criteria

Meta-analysis will be considered only if there is sufficient homogeneity across studies. Specific criteria include a minimum of 5 studies with comparable interventions, populations, and outcomes; use of similar or convertible outcome measures; sufficient data quality (reported means, standard deviations, sample sizes, or extractable effect sizes); and acceptable clinical and methodological heterogeneity.

If meta-analysis is appropriate, the following statistical approaches will be used. Random-effects models will account for between-study variability. Standardized mean differences (SMD) will be calculated for continuous outcomes, while risk ratios or odds ratios will be used for dichotomous outcomes. The I² statistic will quantify heterogeneity, with I² >75% prompting reconsideration of pooling. Funnel plots and Egger’s test will assess publication bias if at least 10 studies are available. Review Manager (RevMan) version 5.4 will be used as the analysis software.

It is important to note that if heterogeneity is too substantial (I² >75%), we will not conduct meta-analysis and will rely on narrative synthesis, thematic synthesis, and framework synthesis to interpret findings.

Missing data will be handled according to established Cochrane guidelines. Study authors will be contacted via email (up to three attempts over a four-week period) to obtain unreported data necessary for meta-analysis. Where data remain unavailable, sensitivity analyses will be conducted to assess the potential impact of missing data on overall findings. Studies with substantial missing data (>20% attrition without adequate intention-to-treat analysis) will be included in narrative synthesis but excluded from meta-analysis, with clear documentation of exclusion rationale.

### Data management procedures

All extracted data will be stored in a secure, password-protected cloud-based repository using institutional data storage systems. Version control will be maintained using unique identifiers and timestamps for all data modifications. Access to the dataset will be restricted to the research team members. Upon publication, a de-identified dataset will be made available through an open-access repository to enhance research transparency and facilitate future meta-analyses. Multiple backup copies will be maintained in different secure locations to prevent accidental data loss. A comprehensive audit trail will document all changes to the dataset throughout the review process.

## DISCUSSION

### Expected outcomes and significance

This systematic review is expected to provide a comprehensive synthesis of evidence on the effectiveness of Problem-Based Learning (PBL) for teaching Evidence-Based Practice (EBP) to nursing and midwifery professionals. It will identify which specific EBP competencies, such as knowledge, attitudes, skills, and behaviors, are most effectively developed through PBL approaches. The review will also characterize the key features of successful PBL interventions, including their duration, intensity, facilitator preparation, and fidelity to core PBL principles.

Furthermore, the review aims to identify barriers and facilitators influencing the implementation of PBL for EBP education across different educational and clinical contexts. By integrating these findings, the review will contribute to evidence-informed curriculum development and educational policy in nursing and midwifery programs globally. Finally, it will highlight existing knowledge gaps and establish priorities for future research in the field of PBL and EBP education.

### Ethical considerations

As a systematic review of published literature, this study does not require ethical approval. Nevertheless, the research team is committed to maintaining the highest standards of research integrity throughout this investigation. This commitment encompasses accurate and transparent reporting of all findings, including any limitations identified during the review process and potential conflicts of interest that may influence the interpretation of results. The team pledges to conduct unbiased selection and analysis of studies, ensuring that inclusion and exclusion decisions are made solely on methodological merit rather than the nature or direction of findings. To prevent any misrepresentation of results, the methodology will be reported with complete transparency, allowing for replication and verification by other researchers. Finally, the research team will provide full acknowledgment of all contributing authors and studies that inform this review, recognizing the scholarly contributions that make this synthesis of knowledge possible.

### Limitations

This systematic review protocol acknowledges several potential limitations that may influence the interpretation of the findings.

*Publication bias*

Publication bias may arise because studies reporting positive results are more likely to be published than those with null or negative findings. To mitigate this risk, the review will incorporate comprehensive grey literature searches, engage with experts to identify unpublished studies, and, where sufficient studies exist, apply funnel plots and statistical tests to assess potential publication bias.

*Heterogeneity in interventions*

PBL interventions differ considerably in their duration, intensity, facilitator training, and adherence to core PBL principles. Such heterogeneity may limit the ability to draw definitive conclusions about the overall effectiveness of PBL. This limitation will be addressed through detailed characterization of interventions, as well as through subgroup and sensitivity analyses.

Variability in EBP competency measurement Differences in the tools used to assess EBP competencies pose another challenge, as these tools may measure varying constructs with different psychometric strengths. This review will prioritize studies that employ validated, standardized EBP assessment instruments. Separate analyses will be conducted for categories of assessment tools, such as self-report versus performance-based instruments and those measuring knowledge, skills, or attitudes. Sensitivity analyses will be performed to evaluate the extent to which measurement approaches influence pooled estimates. Additionally, the psychometric properties of assessment tools used across studies will be documented in detail to support contextual interpretation.

*Limited long-term follow-up*

Educational intervention studies frequently assess outcomes immediately after the intervention, with limited follow-up to determine long-term retention or translation into clinical practice. This review will extract and report all available follow-up time points and will highlight gaps where long-term evidence is lacking.

*Language and temporal restrictions*

Prioritizing English-language publications may introduce selection bias. To partially address this, the review will search five additional regional databases (SciELO, LILACS, CAIRN, BASE, CEEOL) that index non-English studies with English abstracts. Non-English studies will be assessed for eligibility when English abstracts are available, and translation services or multilingual collaborators will be engaged for full-text screening of highly relevant studies. The review will explicitly acknowledge this limitation and will report the number and geographical origins of excluded non-English studies to enhance transparency regarding potential selection bias.

The temporal restriction beginning in 2001 is justified by the period during which EBP principles began formal integration into nursing and midwifery educational frameworks, following developments such as the establishment of the Cochrane Nursing Care Field in 1996 and the emergence of EBP competency frameworks in the early 2000s. This timeframe balances the need for comprehensiveness with contemporary relevance.

*Study design variability*

The inclusion of various study designs (RCTs, quasi-experimental, cross-sectional studies) may introduce methodological heterogeneity that affects the synthesis of results. This will be addressed through rigorous quality assessment and sensitivity analyses.

### Implications for practice and policy

Results will inform evidence-based decisions about curriculum design and teaching methodologies, guide faculty development programs, support integration of effective EBP education strategies, inform accreditation standards and educational guidelines, support resource allocation decisions, and guide development of competency-based education frameworks.

### Expected policy impact and future research directions

If strong evidence supporting PBL effectiveness is found, recommendations may include: integration of PBL approaches into nursing and midwifery education frameworks; revision of accreditation standards to emphasize evidence-based pedagogical approaches; development of faculty training programs for PBL implementation; and resource allocation guidelines for institutions considering PBL adoption.

## CONCLUSIONS

This review will provide evidence on PBL’s effectiveness for EBP education and inform curriculum development and educational policy in nursing and midwifery programs globally. The findings may reveal specific areas requiring further investigation, including optimal PBL implementation strategies, long-term impact assessment, and effectiveness in diverse cultural contexts. Results will be disseminated through peer-reviewed journal publication, presentations at international nursing and midwifery education conferences, educational policy briefs and practice recommendations, and open-access publication to maximize global accessibility.

## Supplementary Material



## Data Availability

Data sharing is not applicable to this article as no new data were created.
