# Supplementary material for: Efficacy of problem-based learning approach for teaching evidence-based practice to midwives and nurses: A systematic review protocol
Source: Eur J Midwifery. 2025 Dec 31;9:10.18332/ejm/215324. doi: 10.18332/ejm/215324 (PMC12817664; doi:10.18332/ejm/215324)
Supplement: Supplementary file 1 [file EJM-9-56-s1.pdf]

APPENDICES

Efficacy of Problem-Based Learning for Evidence-Based Practice teaching to Midwives & Nurses:

A Systematic Review Protocol

APPENDIX 1: COMPLETE DATABASE SEARCH STRATEGIES

This appendix provides complete, reproducible search strategies for all eight databases used in this systematic review. Each strategy includes exact Boolean operators, subject headings, keywords, and limiters to ensure full transparency and enable replication by other researchers.

Table A1.1: PubMed/MEDLINE Search Strategy

Database: PubMed/MEDLINE

Date Range: January 1, 2001 to October 31, 2024

Language: English

Search	Search Terms	Expected Results
1	"Problem-Based Learning"[Mesh] OR "problem-based learning"[Title/Abstract] OR "problem-based learning"[Title/Abstract] OR "PBL"[Title/Abstract] OR "problem-solving learning"[Title/Abstract]	Approx. 45,000
2	"Evidence-Based Practice"[Mesh] OR "evidence-based practice"[Title/Abstract] OR "evidence-based practice"[Title/Abstract] OR "EBP"[Title/Abstract] OR "evidence-based nursing"[Title/Abstract] OR "evidence-based care"[Title/Abstract]	Approx. 180,000
3	"Education, Nursing"[Mesh] OR "Nurse Midwives"[Mesh] OR "nursing"[Title/Abstract] OR "nurse"[Title/Abstract] OR "nurses"[Title/Abstract] OR "midwifery"[Title/Abstract] OR "midwife"[Title/Abstract] OR "midwives"[Title/Abstract]	Approx. 750,000
4	**1 AND 2 AND 3**	Approx. 250-350
5	**4 AND ("2001/01/01"[PDAT]: "2024/10/31"[PDAT])**	Approx. 250-

Search	Search Terms	Expected Results
		350
**6**	**5 AND English[lang]**	**Approx. 230-320**

Final PubMed Search String (copy-paste ready):

(("Problem-Based Learning"[Mesh] OR "problem-based learning"[Title/Abstract] OR "problem based learning"[Title/Abstract] OR "PBL"[Title/Abstract] OR "problem-solving learning"[Title/Abstract]) AND ("Evidence-Based Practice"[Mesh] OR "evidence-based practice"[Title/Abstract] OR "evidence based practice"[Title/Abstract] OR "EBP"[Title/Abstract] OR "evidence-based nursing"[Title/Abstract] OR "evidence-based care"[Title/Abstract]) AND ("Education, Nursing"[Mesh] OR "Nurse Midwives"[Mesh] OR "nursing"[Title/Abstract] OR "nurse"[Title/Abstract] OR "nurses"[Title/Abstract] OR "midwifery"[Title/Abstract] OR "midwife"[Title/Abstract] OR "midwives"[Title/Abstract])) AND ("2001/01/01"[PDAT] : "2024/10/31"[PDAT]) AND English[lang]

Table A1.2: CINAHL Search Strategy

Database: CINAHL via EBSCOhost

Date Range: 2001-2024

S1: (MH "Problem-Based Learning") OR TI ("problem-based learning" OR "problem based learning" OR "PBL") OR AB ("problem-based learning" OR "problem based learning" OR "PBL")

S2: (MH "Evidence-Based Practice") OR TI ("evidence-based practice" OR "evidence based practice" OR "EBP") OR AB ("evidence-based practice" OR "evidence based practice" OR "EBP")

S3: (MH "Education, Nursing+") OR (MH "Nurse Midwives") OR TI (nursing OR nurse OR midwifery OR midwife) OR AB (nursing OR nurse OR midwifery OR midwife)

S4: S1 AND S2 AND S3 *[Limiters: Published Date: 20010101-20241031; Language: English; Peer Reviewed]*

APPENDIX 2: DATA EXTRACTION FORMS

This appendix provides comprehensive data extraction templates that specify all variables to be collected from included studies. These forms ensure systematic and consistent data extraction across all reviewers.

Table A2.1: JBI Data Extraction Form for Experimental/Observational Studies

This comprehensive form covers eight major sections for extracting study characteristics, intervention details, participant information, outcome measures, and results from included comparative studies.

Section 1: Study Identification and Characteristics

Field	Description	Data Type
Reviewer Name	Name of person extracting data	Text
Extraction Date	Date of data extraction	Date
Study ID	Unique identifier assigned	Alphanumeric
Author(s)	First author surname and initials	Text
Publication Year	Year of publication	Year (2001-2024)
Country	Country where study conducted	Text
Funding Source	Source of research funding	Text

***Note:** The complete data extraction form contains eight comprehensive sections covering study identification, design and methodology, participant characteristics, PBL intervention details, comparison interventions, EBP outcomes and measurement tools, additional outcomes, and author conclusions. The full template is available in the study protocol registration on PROSPERO (CRD42023390989).*

Complete Search Strategies: This document contains abbreviated versions of search strategies for brevity. Complete, copy-paste-ready search strategies for all eight databases (PubMed, CINAHL, EMBASE, Web of Science, ERIC, PsycINFO, Cochrane CENTRAL, and Google Scholar) plus grey literature sources are available in the full protocol manuscript and PROSPERO registration.

Complete Data Extraction Forms: The abbreviated tables in this appendix demonstrate the structure and detail level of our data extraction templates. Complete forms covering all eight sections for quantitative studies and comprehensive qualitative data extraction templates are available in the full protocol and PROSPERO registration to ensure complete reproducibility.

© 2025 Komuhangi G. et al.
